# Can portable glucose and lactate meters be a useful tool in quantifying stress of juvenile Chinook salmon?

**DOI:** 10.1093/conphys/coad046

**Published:** 2023-07-06

**Authors:** Benjamin M Vaage, Stephanie A Liss, Eric S Fischer, Fenton Khan, James S Hughes

**Affiliations:** Pacific Northwest National Laboratory, 902 Battelle Blvd, Richland, WA, United States, 99354; Pacific Northwest National Laboratory, 902 Battelle Blvd, Richland, WA, United States, 99354; Pacific Northwest National Laboratory, 902 Battelle Blvd, Richland, WA, United States, 99354; U.S. Army Corps of Engineers, 333 SW 1st Ave, Portland, OR, United States, 97204; Pacific Northwest National Laboratory, 902 Battelle Blvd, Richland, WA, United States, 99354

**Keywords:** stress, portable meters, lactate, glucose, Chinook salmon

## Abstract

Blood plasma analyses can provide researchers, aquaculture facilities and fisheries managers with valuable insights into the physiological state and welfare of fish. For example, glucose and lactate are part of the secondary stress response system, and elevated concentrations are indicators of stress. However, analysing blood plasma in the field can be logistically difficult and typically involves sample storage and transport to quantify concentrations in a laboratory setting. Portable glucose and lactate meters offer an alternative to laboratory assays and have shown to be relatively accurate in fish, but these tools have only been validated for a few fish species. The objective of this study was to investigate if portable meters could be reliably used in Chinook salmon (*Oncorhynchus tshawytscha*). As part of a larger stress response study, juvenile Chinook salmon (157 ± 17 mm fork length [mean ± standard deviation; SD]) were exposed to stress-inducing treatments and sampled for blood. Laboratory reference glucose concentrations (milligrams per deciliter; mg/dl; *n* = 70) were positively correlated with the Accu-Check Aviva meter (Roche Diagnostics, Indianapolis, IN) measurements (*R^2^* = 0.79), although glucose values were 1.21 ± 0.21 (mean ± SD) times higher in the laboratory than with the portable meter. Lactate concentrations (milliMolar; mM; *n* = 52) of the laboratory reference were also positively correlated (*R^2^* = 0.76) with the Lactate Plus meter (Nova Biomedical, Waltham, MA) and were 2.55 ± 0.50 times higher than portable meter. Our results indicate both meters could be used to measure relative glucose and lactate concentrations in Chinook salmon and provide fisheries professionals with a valuable tool, particularly in remote field settings.

## Teaser

The ability to quantify stress responses, such as glucose and lactate, in situ could aid in the recovery of threatened or endangered species, like Chinook salmon (Oncorhynchus tshawytscha). Portable meters used to measure glucose and lactate concentrations could be used with Chinook salmon, but first must be validated for accuracy.

## Introduction

Fish experience stress in their environment from a variety of sources, which can be real or perceived threats, and physical or chemical in nature, such as the presence of predators or poor water quality (Barton, 2002; Schreck and Tort, 2016). A primary physiological response to stress in most vertebrates is increased circulating cortisol concentrations in the blood plasma, which can be used to quantify the severity and duration of a stressor (Barton and Iwama, 1991; Baker and Vynne, 2014; Schreck and Tort, 2016). Physiological responses to stress aid in the process of generating the necessary energy and oxygen needed to survive the stressor (Reid *et al.,* 1998; Schreck and Tort, 2016). Although stress is a natural and necessary response, severe acute or chronic exposure can compromise fish health. Depending on the severity and duration of the stressor, energy may be diverted to combating the stressor from areas such as growth, reproduction and disease resistance, which can reduce the likelihood of survival (Van Weerd and Komen, 1998; Schreck and Tort, 2016). Insight into the stress levels (i.e. plasma cortisol concentrations) of fish can help fisheries managers make informed decisions about increasing survival of wild fishes or best rearing practices for cultured species. Measuring plasma cortisol concentrations involves blood sampling, centrifuging, cold storage and technical laboratory assays. Although cortisol concentrations are a reliable method for quantifying stress in fish, the process of evaluating cortisol concentrations could be resource- and time-consuming, and field-practical alternatives may be available.

Glucose and lactate are secondary stress response metabolites that can also be effective at measuring stress levels in fishes (Grutter and Pankhurst, 2000; Warriner *et al.,* 2020; Herrera *et al.,* 2023; Samaras *et al.,* 2023). Portable glucose and lactate meters designed for mammals (i.e. humans) are available and have the potential to be used with fish in a similar manner, which could provide biologists with *in situ* knowledge of the physiological state of a fish. Furthermore, and unlike the process of evaluating cortisol concentrations, portable glucose and lactate meters are small, lightweight, deliver fast results and could eliminate the need of sample transportation and storage. However, stress responses are different among species and life stages; therefore, individual species or strains within a species should be examined separately (Barton, 2000; Awruch *et al.,* 2011; Schreck and Tort, 2016). Previous research validating portable glucose and lactate meters in fish are limited to only a few species. Beecham *et al.* (2006) measured glucose and lactate concentrations of fingerling channel catfish (*Ictalurus punctatus*) with handheld meters and laboratory assay kits. The authors determined the meters underestimated both glucose and lactate levels compared with a laboratory reference but could still be used reliably for relative concentrations. Similar studies evaluating rainbow trout (*Oncorhynchus mykiss*) and Atlantic cod (*Gadus morhua*) showed comparable findings, with meters underestimating concentrations compared with laboratory methods (Wells and Pankhurst, 1999; Brown *et al.,* 2008). To our knowledge, research validating the accuracy of portable meters to measure glucose and lactate in Chinook salmon (*Oncorhynchus tshawytscha*) has not been conducted.

Currently, six evolutionary significant units or distinct population segments of Chinook salmon in the Pacific Northwest, USA, are listed as endangered or threatened and protected under the Endangered Species Act (National Marine Fisheries Service (NMFS, 2008)). Adding new tools that provide insight into the physiological state of individual fish and how it relates to their environment can ultimately aid in recovery efforts by providing additional metrics for managers of threatened species, particularly for species of concern like Chinook salmon (Gosselin *et al.,* 2022). The objective of this research was to investigate if portable meters have potential as *in situ* field tools in reliably measuring glucose and lactate levels in juvenile Chinook salmon. Our goal was to compare the meter concentrations with laboratory reference concentrations (i.e. laboratory assay kits) and examine any possible relationships. If proven reliable, the meters could provide an additional tool for fisheries researchers and managers. The meters used herein are commercially available and were appropriate for this study based on availability (i.e. in stock), portability (light weight) and cost. Other similar meters have also been used in recent studies (Beecham *et al.,* 2006; Brown *et al.,* 2008; Stoot *et al.,* 2014; Ball and Weber, 2017). The use of the meters in this study is solely intended for research purposes and to identify their potential as viable tools for field studies.

## Methods

### Fish husbandry and sampling

Fish care and use for this study were approved by the Pacific Northwest National Laboratory (PNNL) Institutional Animal Care and Use Committee (protocol no. 2020–06). Juvenile Chinook salmon (*n* = 70) approximately age-1 (157 ± 17 mm in fork length [mean ± standard deviation; SD]) were provided by the Oregon State University Wild Fish Surrogate Program. These 70 individuals were a subset of fish that were exposed to dam passage simulations and sampled for a larger fish passage stress response study (Liss *et al.,* 2022). The study site was Green Peter Dam, located on the Middle Santiam River near Sweet Home, Oregon. All fish were housed in 400-l tanks with a flow rate of one tank volume per hour to maintain water quality before sampling. Tank temperatures were 9.5–15.8°C (mean: 12.1°C), and dissolved oxygen (DO) ranged from 7.8 to 9.2 mg/l (mean: 8.6 mg/l). Fish were exposed to one of two types of simulated dam passage treatments or were control fish. The two treatments were transporting fish downstream of the dam by truck conveyance or through a bypass pipe that was designed to pass out-migrating salmonids (elevation drop ~91 m) through the dam. After the treatments, fish were recollected in a tank at a fish collection facility at the base of the dam. Collection tank temperatures were 10.3–15.6°C (mean: 12.1°C), and DO was 7.4–9.3 mg/l (mean: 8.6 mg/l).

Subsamples of fish were taken at six sampling time intervals (0, 0.5, 1, 3, 6, 12, and 24 h) within a 24-h post-treatment period. To sample fish, individuals were placed into 5 l of water dosed with a lethal concentration of anesthesia (250 mg/l MS-222) for 30 s (i.e. until sedation). Once immobilized, a minimum of 0.4 ml of whole blood was removed from the fish via caudal venipuncture using 21-gauge needles (BD, Franklin Lakes, NJ) pre-rinsed with a heparin sodium solution (1000 units heparin to 1 ml water) to prevent blood clotting (Houston, 1990). A small amount of whole blood was reserved in the syringe for on-site glucose and lactate measurements immediately after blood sampling. The rest of the whole blood was spun in a centrifuge (model HS120301; Heathrow Scientific, Vernon Hills, IL) at 6000 rpm for 4 min to separate red blood cells from plasma. Plasma was extracted, divided equally and placed into two individually labeled microcentrifuge tubes using disposable pipettes. Samples were immediately put into a −20°C freezer until they could be moved to and stored at PNNL in a −80°C freezer, where they remained until laboratory analysis. Study fish were euthanized after blood sampling.

### Glucose and lactate meter sampling

Glucose (milligrams per deciliter; mg/dl) was measured using the Accu-Check Aviva meter (Roche Diagnostics, Indianapolis, IN). The meter had a test range of 20–600 mg/dl, and the associated test strips contained the enzyme glucose dehydrogenase, which converts glucose in the blood to gluconolactone, and measured glucose as a plasma value. Lactate (milliMolar; mM) was measured using the Lactate Plus Meter (Nova Biomedical, Waltham, MA). The range of detection was 0.3–25.0 mM, and the meter measured blood samples using a lactate oxidase biosensor that determines lactate concentration as a plasma value. Both meters were calibrated daily using the protocols provided by the manufacturers before analysing fish blood.

### Glucose and lactate laboratory reference assays

Laboratory glucose analyses were conducted using the QuantiChrom Glucose Assay Kit (DIGL-100; BioAssay Systems, Hayward, CA). The kit uses the *o*-toluidine method and measures absorbance at 630 nm to estimate glucose concentration of the sample. Optical density (OD) of the sample was subtracted from the OD of the blank (water) standard and divided by the slope of the standard curve line to calculate concentration of each sample. Laboratory lactate analyses were conducted using the EnzyChrom L-Lactate Assay Kit (ECLC-100; BioAssay Systems). The light intensity of the sample was measured at 565 nm and is directly proportionate to lactate concentration. Because samples potentially contained endogenous enzyme activity, two reactions were conducted: one with an enzyme and one without (control). Following the lactate kit protocol, all samples were diluted (6-fold) and concentrations were calculated as the difference between OD of the reaction with the enzyme and the reaction without and divided by the slope of the standard curve. Because the samples were diluted 6-fold, final lactate concentrations were determined by back calculation of multiplying by 6-fold. All samples and standards were run in duplicate using the BioTek model EON photospectrometer (Agilent Technologies Inc., Santa Clara, CA).

### Statistical analysis

Glucose and lactate reference intra-assay coefficients of variation (*CV*) between replicate samples were calculated in the software program Gen5 (v. 2.09.2, Agilent Technologies Inc.) using the following equation:

$$ CV=\left(\frac{standard\ deviation}{mean}\right)\times 100. $$

Laboratory reference sample and standard replicate *CV*s of ≤5% were included in the glucose analyses. Because lactate assays involved repeated measurements (i.e. 4 per sample), samples with at least 75% (3 of 4) showing ≤5% *CV* were used for analyses. In addition, only sample glucose and lactate reference concentrations that fell within their respective standard curve range were included in the analyses. Subsequently, mean values of sample and standard assay replicates were used when *CV* guidelines were met.

Laboratory sample concentrations were plotted against corresponding meter concentrations to examine potential relationships using simple linear regression and coefficient of determination values (i.e. *R^2^*). In addition, absolute differences between laboratory and meter concentrations were plotted against the mean of the two corresponding measurements to examine the heteroscedasticity and associated Pearson correlation coefficient, *R* (Lindholm and Altimiras, 2016). Finally, laboratory reference and meter concentrations were plotted by individual samples, with a line of best fit generated by measurement type to visually examine differences in concentration and slope of line. Analyses were performed in R with a significance level set to *α* = 0.05 (R Core Team, 2021).

## Results

### Glucose

**Figure 1:**
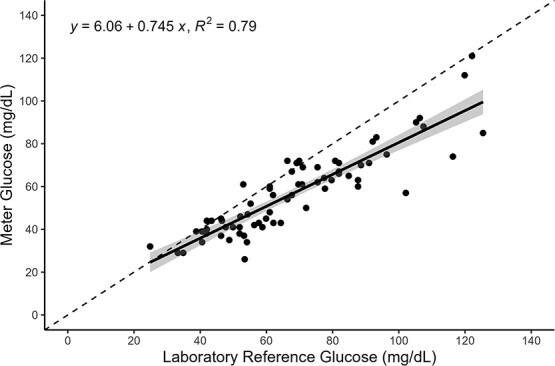
Measured glucose meter concentrations (mg/dl; *y*-axis) plotted against laboratory reference glucose concentrations (mg/dl; *x*-axis) of corresponding Chinook salmon samples. Dashed line illustrates a 1:1 relationship as reference. Circles represent individual fish and their corresponding glucose concentrations, with a linear regression (solid black line with 95% confidence interval shaded around the line) fit to the data.

**Figure 2:**
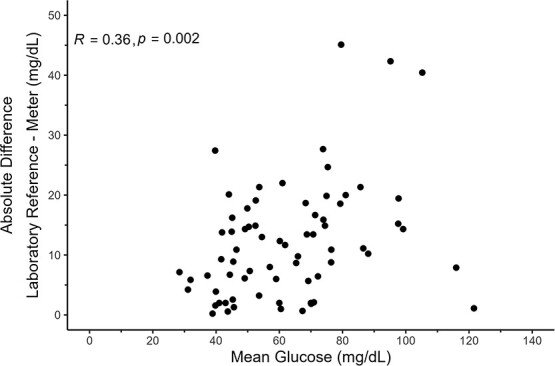
Absolute differences between meter and laboratory reference glucose (mg/dl; *y*-axis) plotted against the mean glucose (mg/dl; *x*-axis) of the corresponding samples. Pearson correlation coefficient, *R* and associated *P* value are also shown.

**Figure 3:**
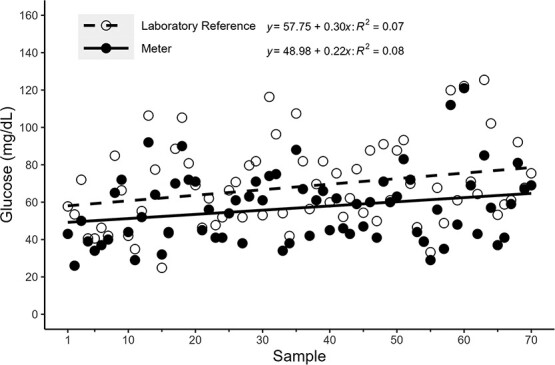
Glucose concentration (mg/dl: *y*-axis) of individual fish samples by measurement type. Individual fish (Sample; *x*-axis) are represented by paired circles (open and closed). Open circles (*n* = 70) and the dashed line depict glucose concentrations for laboratory reference fish, whereas black circles (*n* = 70) and the solid line depict the glucose meter concentrations.

All (*n* = 70) laboratory glucose replicates had <5% *CV* (1.5 ± 1.2 [mean ± SD]) and were used in the analyses. The standard curves of the laboratory glucose assays showed a high goodness-of-fit (*R^2^* = 0.99 ± 0.001). A simple linear regression analysis showed a positive correlation with an *R^2^* = 0.79 and *P* < 0.001 (Figure 1). Absolute differences between measurement types and mean concentration of the corresponding samples generated a Pearson correlation coefficient of *R* = 0.36 and *P* = 0.002, indicating unequal variance (Figure 2). Overall, the laboratory reference glucose concentrations measured ~1.2 times higher than the concentrations of the portable meter (1.21 ± 0.21; Figure 3).

### Lactate

**Figure 4:**
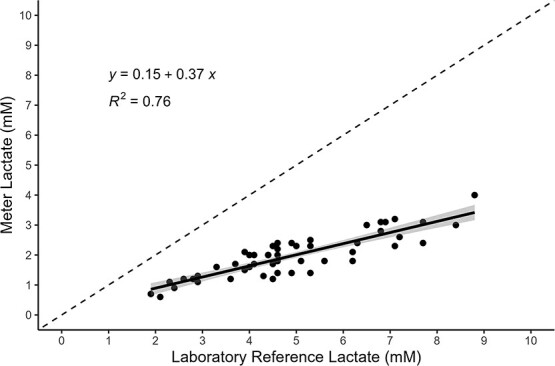
Measured lactate meter concentrations (mM; *y*-axis) plotted against the laboratory reference lactate concentrations (mM; *x*-axis) of corresponding Chinook salmon plasma samples. Dashed line illustrates a 1:1 relationship as reference. Circles are individual fish lactate concentrations with a linear regression (solid black line with 95% confidence interval shaded around the line) fit to the data.

**Figure 5:**
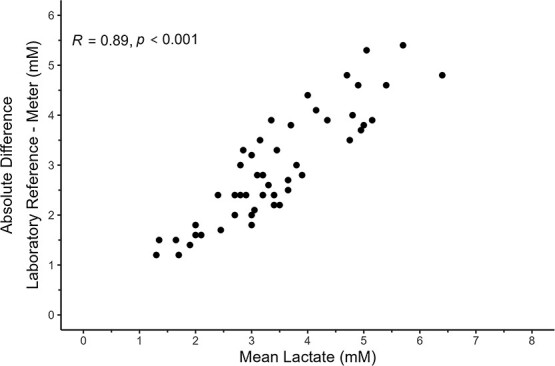
Absolute differences between meter and laboratory reference lactate (mM; *y*-axis) plotted against the mean lactate (mM; *x*-axis) of the corresponding samples. Pearson correlation coefficient, *R* and associated *P* value are also shown.

**Figure 6:**
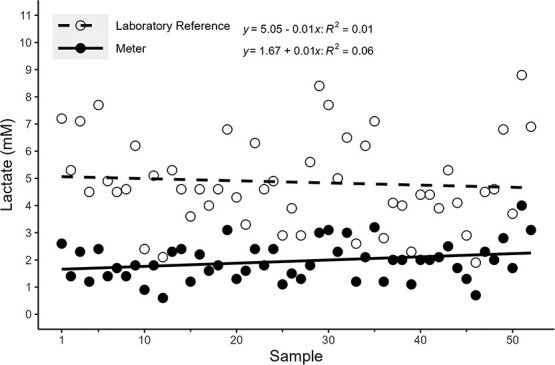
Lactate concentrations (mM; *y*-axis) of individual sample by measurement type. Individual fish (Sample; *x*-axis) are represented by paired circles (open and closed). Open circles (*n* = 52) and the dashed line depict lactate concentrations for laboratory reference fish, whereas black circles (*n* = 52) and the solid line depict lactate meter concentrations.

All 70 samples were diluted 6-fold, but six samples fell outside the standard curve range, whereas another 12 failed to meet the *CV* threshold criteria; therefore, these 18 samples were excluded from analyses. A total of 52 laboratory samples met the 5% *CV* and standard curve range criteria for lactate and were subsequently used in the analyses. The standard curves of the lactate assays showed a high goodness-of-fit (*R^2^* = 0.99 ± 0.007). A simple linear regression analysis showed a positive correlation between the laboratory reference and portable meters with an *R^2^* = 0.76 and *P* < 0.001 (Figure 4). Absolute differences between measurement type and mean lactate (mM) of corresponding samples generated a Pearson correlation coefficient of *R* = 0.89 and *P* < 0.001, showing increasing variance with increasing mean concentrations (Figure 5). Laboratory reference lactate concentrations were ~2.6 times higher than the concentrations of the portable meter (2.55 ± 0.50; Figure 6).

## Discussion

This study successfully compared portable glucose and lactate meters with reference laboratory concentrations. Both meters underestimated concentrations compared with laboratory references; however, consistent positive correlation was observed with both meters. Although positive correlations were measured, the use of generated linear equations as a method to correct meter inaccuracies is not recommended due to the observed heteroscedasticity in the higher concentration ranges (Figures 2 and 5). Rather, the utility of each of the meters remains as a field tool to detect relative changes *in situ* with juvenile Chinook salmon. Meter performances for adult Chinook salmon are unknown and should be validated before use because meter concentrations may vary with different life stages, although they may have the same potential utility as with juveniles.

Measured *R^2^* values fell below 0.80 and, when compared with other similar studies, could be viewed as marginal (Wells and Pankhurst, 1999; Beecham *et al.,* 2006; Brown *et al.,* 2008). For example, multiple studies measured higher levels of correlation (*R^2^* > 0.90) and accuracy but involved different species such as Atlantic cod, Nile tilapia (*Oreochromis niloticus*) and bonefish (*Albula vulpes*), while testing different meter brands than the ones used for this study (Evans *et al.,* 2003; Brown *et al.,* 2008; Cooke *et al.,* 2008). In contrast, Ball and Weber (2017) validated a handheld glucose meter on fingerling walleye (*Sander vitreus)* and measured an *R^2^* = 0.71, leaving potential use of meters based on *R^2^* values open to interpretation.

One of the few studies that evaluated glucose and lactate meters (albeit using different meters) in a salmonid species compared glucose and lactate meters with reference laboratory assays in rainbow trout (Wells and Pankhurst, 1999). Wells and Pankhurst (1999) found their glucose meter underestimated by ~10%, with an *R^2^* = 0.72 (presented as Pearson correlation *R* = 0.85), whereas the reference lactate concentrations were nearly three times that of the meter, with an *R^2^* = 0.69 (presented as Pearson correlation *R* = 0.83). Interestingly, the Wells and Pankhurst (1999) results exhibited comparable underestimation and correlation to our study in both glucose and lactate meters. Although our data followed similar trends to those observed in other studies (meter concentration underestimation and positive correlation), the underlying mechanisms involved in these trends and our measured results remain unknown but may be attributed to the meters being designed for human blood and the differences that exist between fish and humans (e.g. cold-blooded vs. warm-blooded as it relates to whole-blood temperature) (Stoot *et al.,* 2014).

Multiple factors could contribute to the lack of accuracy of our study meters. First, both meters are designed to convert whole-blood sample concentrations to plasma concentrations using equations derived for humans. These correction factors are based on human blood and may not readily translate to Chinook salmon blood, potentially due to differences in temperature (Stoot *et al.,* 2014). Fish in our study were collected from water ~12°C, whereas human blood is typically ~37°C. Second, handheld meters test whole blood, whereas the laboratory assays measure plasma values. Studies correlating glucose and lactate whole blood to plasma concentrations in fish report high agreement, but generally whole-blood concentrations are lower (Iwama *et al.,* 1995; Wells and Pankhurst, 1999; Serra-Llinares *et al.,* 2012). In addition, plasma samples are commonly stored between −20 and −80°C, often for prolonged periods, whereas whole-blood measurements are typically taken immediately after blood is drawn. Although the meter and laboratory reference testing methods differ, the effects of cold storage may not be significant. For example, Serra-Llinares *et al.* (2012) measured lactate concentrations of fresh whole blood and frozen (−40°C) then thawed plasma lactate concentrations of Atlantic cod. The frozen and thawed plasma lactate (mM) were highly correlated (*R^2^* = 0.99) to whole-blood lactate (mM) and measured significantly higher than blood. Interestingly, whole-blood lactate (mM) was 8% lower than plasma lactate (mM) at ≤4 mM and 17.5% lower from 4 to 10 mM, a pattern also observed in our research (Serra-Llinares *et al.,* 2012). Regardless of the underlying reason for the underestimation of the portable meters, the potential utility of a relative comparison still proves valuable and diverse.

Both meters can be used as field tools for measuring fish stress but are not limited to that alone. For example, glucose meters could be used to evaluate the smoltification process of juvenile salmonids and their readiness to migrate because blood glucose increases as fish transition from parr to smolt (Wedemeyer *et al.,* 1980; Gosselin *et al.,* 2022). In addition, lactate meters designed for humans are commonly used in sports medicine to measure and track performance (Pyne *et al.,* 2000). This application could be transferred to fish and used to examine performance gains of exercise-trained cultured fishes that are used to supplement threatened wild stocks. When conducting field research, a commercially available tool that provides immediate results, convenience, portability and has a low cost when compared with laboratory procedures involving high-tech equipment and expensive assay kits may be worth the trade-off if a relative concentration ranking is sufficient to gain prudent information.

Ultimately, the use of these tools depends on specific research needs. As technology advances and biologists gain more understanding of fish physiology, new tools may arise that can be used across whole taxonomic groups, and for now, devices like the meters tested in this study should be validated on a species-by-species basis to best understand the potential differences in meter readings for a given species (Barton, 2000; Awruch *et al.,* 2011; Schreck and Tort, 2016). The authors suggest that if relative concentrations are satisfactory for research objectives, both meters will provide valuable information for juvenile Chinook salmon, especially in the field or in a laboratory when rigorous and extensive sample analysis may not be feasible. Furthermore, the scope of use may extend beyond secondary stress response metrics and could be used as novel instruments in different types of physiological research.

## Data Availability

The data that support the findings of this study are available on request from the corresponding author (B.M.V.).
